# Team‐level identification predicts perceived and actual team performance: Longitudinal multilevel analyses with sports teams

**DOI:** 10.1111/bjso.12277

**Published:** 2018-09-21

**Authors:** William E. Thomas, Rupert Brown, Matthew J. Easterbrook, Vivian L. Vignoles, Claudia Manzi, Chiara D'Angelo, Jeremy J. Holt

**Affiliations:** ^1^ University of Sussex Falmer UK; ^2^ Catholic University of the Sacred Heart Milan Italy; ^3^ Centre for Team Excellence Steyning UK

**Keywords:** social identity, team performance, multilevel modelling, group processes, sports teams

## Abstract

Social identification and team performance literatures typically focus on the relationship between individual differences in identification and individual‐level performance. By using a longitudinal multilevel approach, involving 369 members of 45 sports teams across England and Italy, we compared how team‐level and individual‐level variance in social identification together predicted team and individual performance outcomes. As hypothesized, team‐level variance in identification significantly predicted subsequent levels of both perceived and actual team performance in cross‐lagged analyses. Conversely, individual‐level variance in identification did not significantly predict subsequent levels of perceived individual performance. These findings support recent calls for social identity to be considered a multilevel construct and highlight the influence of group‐level social identification on group‐level processes and outcomes, over and above its individual‐level effects.

## Background

Teams and groups form the foundations of human society. From the workplace to space exploration, teams are essential features of human social organization and are at the forefront of many human accomplishments. Hence, understanding what drives high *team* performance is crucial to a multitude of societal, sporting, and organizational functions. Yet, it is notable that both lay accounts and psychological research often have a strongly individual‐centred perspective when attempting to understand team performance (Baumeister, Ainsworth, & Vohs, 2015; Brown, 2016; Kozlowski & Ilgen, 2006; Nielsen, Hrivnak, & Shaw, 2009).

In the social identity literature, identification has usually been treated as an individual‐level variable, especially in relation to performance (although see Dietz, van Knippenberg, Hirst, & Restubog, 2015; Van Der Vegt & Bunderson, 2005). While some individuals will identify more than others with any given group, it is also true that some groups tend to arouse identification more than others. This can be due to a number of factors that occur at the group level*,* such as sharing of information, team climate, shared norms, and effects of leadership (Fransen *et al*., 2015; Kerr, Aronoff, & Messé, 2000; Kerr & Hertel, 2011; Postmes, Haslam, & Swaab, 2005; van Dick, Grojean, Christ, & Wieseke, 2006). Thus, studying the influence of individual differences and team differences in social identification together is likely to give a more complete understanding of team performance.

In the present paper, we seek to examine how social identification as a *multilevel* construct – distinguishing between *individual‐level identification* (ILI) and *team‐level identification* (TLI) – impacts on team performance outcomes. ILI refers to within‐team differences between individuals in levels of social identification. TLI refers to systematic differences between teams in levels of social identification (i.e., variance in identification that is attributed to differences between teams, rather than between individuals).

### Multilevel nature of social identification

Current conceptualizations of social identification typically state that identification occurs when an *individual* strives to attach themself to a social group (i.e., ILI, see Haslam, 2014; Hogg, van Knippenberg, & Rast, 2012). Although this conceptualization assumes that ILI is, at least to some extent, derived from group‐level processes, it essentially treats social identification as an individual difference variable rather than a property of the group concerned (Ashmore, Deaux, & McLaughlin‐Volpe, 2004). When group‐level identity constructs have been studied, these have typically been done in isolation from ILI (Dietz *et al*., 2015; Van Der Vegt & Bunderson, 2005). Statisticians have argued that studying group processes in general necessitates a multilevel approach (Hoffman, 2015; Hox, 2010). This is because individuals are influenced by group factors such size or status and groups are formed of individuals. Seen in this way, within‐group (individual‐level) and between‐group (team‐level) factors should be considered together when researching group processes (Arrow, McGrath, & Berdahl, 2000).

Recently, researchers have begun to untangle these two levels of analysis by treating social identification as a multilevel construct (Jans, Leach, Garcia, & Postmes, 2015; Ozeki, 2015). By decomposing observed individual responses into individual‐level and group‐level components, researchers have distinguished between effects that should be attributed to individual differences and processes and those that should be attributed to group‐level differences and processes. TLI (also described as ‘group‐level group identity’, Ozeki, 2015) refers to the variance in social identification that can be attributed to the team level. Treated in this way, TLI represents the emergent identity of the group or team, rather than the intrapsychic processes of each separate individual (i.e., ILI) (Khan *et al*., 2015). Put simply, TLI captures the influence of the team on levels of social identification.

Ozeki (2015) found that TLI was an essential element in group formation and had a positive effect on interactions, emotional bonds, and interdependence among group members. Recent longitudinal multilevel analyses confirmed that identification is more than an individual difference and is, at least partly, based on group‐level processes (Jans *et al*., 2015; Thomas *et al*., 2017). Notably, Jans *et al*. (2015) also found that, for face‐to‐face groups, the influence of the group on identification increases as a function of interaction among group members.

The development of group differences in TLI over time may occur through two separate but related processes – group ‘consensualization’ and/or group ‘polarization’. Group *consensualization* entails that, as members interact, they become increasingly similar – whether high or low – in their levels of identification, which constitutes higher or lower TLI (Haslam, Turner, Oakes, McGarty, & Reynolds, 1997). On the other hand, group *polarization* entails that groups, as a whole, become increasingly different from one another in their levels of identification (Turner, 1991). Jans and colleagues found some support for both consensualization and polarization in levels of identification, but their results were inconsistent across different kinds of group studied. Ozeki (2015) and Jans *et al*. (2015) focused on artificial groups or study groups at University, and so there is a need to explore other types of real‐life groups. Nevertheless, this research does suggest that social identification is more than an idiosyncratic personal representation of the group but instead occurs at *both* an individual level (ILI) and a team level (TLI).

### TLI and team performance

To our knowledge, only a few studies have investigated the association between TLI and team performance (Dietz *et al*., 2015; Solansky, 2011; Van Der Vegt & Bunderson, 2005; van Dick *et al*., 2006). In two longitudinal studies, Solansky (2011) found that teams with high TLI performed better than teams with low TLI. However, despite the longitudinal multilevel nature of the data collected, Solansky only reported a correlation between TLI and team performance at a single level of analysis. Thus, it is difficult to draw inferences about the respective roles of ILI and TLI.

More recently, Dietz *et al*. (2015) found that TLI motivated team performance among people with what they called ‘performance‐prove goal orientation’ (people who tend to focus on performance‐related outcomes). Conversely, when TLI was low, ‘performance‐prove goal orientation’ motivated individual‐level performance. This suggests that, for people who are driven to achieve performance goals, a high TLI focuses their performance orientation at the team level. However, this study only investigated TLI (not ILI) and used a cross‐sectional design, making it difficult to draw conclusions regarding the direction of relationships. One previous study has demonstrated a directional relationship between a group‐level identity and performance. van Dick *et al*. (2006; Study 2) found a small, marginally significant lagged relationship between prior organizational identity and subsequent organizational citizenship behaviour (e.g., helping colleagues and making innovative suggestions). While this hints at the importance of group‐level identity to performance outcomes, the authors’ focus was solely at the group level and on organizational, rather than team, identity (which may be a different construct: van Knippenberg & van Schie, 2000).

It is worth noting here that, in contrast to the lack of research on TLI, group cohesion is widely studied within the team literature and linked with team performance (Beal, Cohen, Burke, & McLendon, 2003; Casey‐Campbell & Martens, 2009; Castaño, Watts, & Tekleab, 2013; Kozlowski & Ilgen, 2006; Mullen & Copper, 1994; Rovio, Arvinen‐Barrow, Weigand, Eskola, & Lintunen, 2010). Cohesion is variously defined, but one prominent definition is ‘group members inclination to forge social bonds, resulting in members sticking together and remaining united’ (Carron, 1982). The main difference between cohesion and social identification is that cohesion has traditionally been understood in terms of interpersonal attraction among group members, rather than as a genuine group‐level construct (Hogg, 1992).

Hogg (2015) sought to provide an alternative perspective on cohesion with his self‐catergorization account that defined it in terms of group members’ attraction to the notion of the group. Based on Hogg's idea that groups that have a strong group identity are more cohesive, Postmes, Spears, and Cihangir (2001) even used group means of group identification as a measure of group cohesiveness. In multilevel modelling, such group means should be treated as group‐level variable (and thus akin to TLI, see Ozeki, 2015). Overall, then, although theory and tentative research findings hint that TLI may be an important predictor of team performance, there is a paucity of robust empirical research in this area.

### The present study

Our current study had two main aims: (1) to investigate the directionality of effects between TLI and team‐level performance and (2) to explore whether these effects are irreducible to those of ILI on individual‐level performance (i.e., effects of TLI on performance outcomes are over and above what would be expected simply by aggregating the effects of ILI on individual performance outcomes). Although our main focus was on predicting performance, our design also allowed us to quantify the extent of systematic variance in TLI among sports teams and to investigate the underlying processes of consensualization (when members become increasingly similar in their levels of identification) and polarization (when teams, as a whole, become increasingly different from one another in their levels of identification).

To address these aims, we conducted longitudinal research with 45 sports teams from England and Italy over a 6‐month period with four time points. These sports teams contain established and new group members, have a team history and future, contain a team leader (team captain), and compete on a regular basis. They are, therefore, meaningful social groups with parallels to many other kinds of groups across various situations. For example, team members also interact outside of sporting functions, with social activities held throughout the year. Accordingly, for some members, the sports team they join can form an integral part of daily life.

We take a multilevel analytical approach that enables us to decompose identification and performance into within‐team and between‐team variance. As shown in Table 1, this decomposition allows us to test predictions of five performance outcomes:

**Table 1:** Individual and team performance outcomes

	Perception of individual performance	Perception of team performance	Actual performance
Individual level	Perceived individual performance (within‐team variance): *Variance in perceived individual performance attributed to the individual level*	Perceived team performance (within‐team variance): *Variance in individual rating of team performance attributed to the individual level*	No measure available
Team level	Perceived individual performance (between‐team variance): *Variance in perceived individual performance attributed to the team level*	Perceived team performance (between‐team variance): *Variance in individual rating of team performance attributed to the team level*	Actual team performance: *Standardized team scores*

#### Perceived individual performance (within‐team variance)

This refers to the component of variance in individuals’ ratings of their own performance that can be attributed to individual differences. This was considered our main outcome variable for individual performance.

#### Perceived individual performance (between‐team variance)

This refers to the component of variance in individuals’ ratings of their own performance that can be attributed to differences between teams – thus representing systematic effects of team membership on individuals’ (self‐rated) performance. Note that the target for evaluation in this measure is still the individual's personal performance, and this measure does not take into consideration how members perceive the team as a whole as performing.

#### Perceived team performance (within‐team variance)

This refers to the variance in individuals’ ratings of their team's performance that can be attributed to individual differences. Since a team cannot be considered to have performed well solely on the basis of one member's perception of team performance, this was not considered one of our primary performance outcomes.

#### Perceived team performance (between‐team variance)

This refers to the variance in members’ ratings of team performance that can be attributed to differences between teams. As this concerns the team performance as perceived by multiple team members, we consider this variable to be the best subjective estimate of how the team performed.

#### Actual team performance

Due to positivity bias, perceptions of team performance may not necessarily reflect accurately how well the team actually performed. Thus, we sought to gain a measure of actual team performance. By standardizing team score differences within the 14 different sports in our sample, we were able to achieve an actual performance measure that is comparable across our sample (Smith, Bellamy, Collins, & Newell, 2001; Wolfe & Box, 1987, for similar analyses). Consequently, although actual performance could not be measured at two levels (there was no measure of actual individual performance), we are able to explore how TLI relates to actual team performance while accounting for the multilevel nature of our team identity data.

#### Hypotheses

Based on the above theoretical reasoning, we hypothesized that TLI would predict perceived (H1) and actual team performance (H2), as well as team‐level variation in perceived individual performance (H3). Moreover, we hypothesized that TLI would predict team‐level variation in perceived team or individual performance, over and above any aggregated individual‐level effects of ILI (H4). Thus, we expected that TLI would predict all team‐level performance outcomes and that these effects would not be reducible to effects of ILI on individual‐level performance outcomes.

## Method

### Participants and design

Participants were approached during team training sessions and asked to complete a short questionnaire on team psychology. The questionnaire also included items that were relevant to another study that investigated identity motives in group situations and was not related to performance (Thomas *et al*., 2017). Four hundred and one team members completed the questionnaire on at least one time point. We excluded 31 participants who only completed one wave and one participant who reported belonging to a team that included only himself (male trampolining team). This left a total of 369 participants clustered within 45 teams. One hundred and eighty‐eight were from a university on the south coast of England (106 men, *M* = 20.80 years, *SD* = 2.63; and 82 women, *M* = 20.27 years, *SD* = 1.75); the remaining 181 were from recreational sports teams in Italy (100 men, *M* = 22.52 years, *SD* = 7.01; and 81 women, *M* = 22.85 years, *SD* = 6.77). Both the English and Italian teams would typically have one training session and one match per week.

A total of 1,202 occasions of data were collected across all four time points (T0 = 312, T1 = 290, T2 = 309, and T3 = 291) with 274 missing occasions. Participants were from 14 different sports (basketball, hockey, netball, fencing, tennis, football, volleyball, trampolining, ultimate Frisbee, badminton, water polo, synchronized swimming, swimming, and cycling), which comprised 45 different teams (*M*_size_ = 8.2, *SD*_size_ = 3.54). Thus, we had a clustered longitudinal design, with individuals nested within teams over time. It should be noted that teams playing ‘individual’ sports (e.g., tennis and trampolining) still behaved as teams. They trained together, socialized together, travelled to matches together, and won and lost *as a team,* rather than individuals.

### Procedure

The four waves of data collection took place for both the English and Italian samples over a 6‐month period during the same sports season from the beginning of October 2014 to mid‐March 2015. To allow team members to be stably allocated, the initial data collection for the English sample took place 2 weeks into the academic term. Data collection took place at approximately 8‐week intervals and at identical time periods for both samples. Once participants had completed the questionnaire, they were given a small confectionary item and thanked for their time.

### Measures

Social identification with the team was recorded using a six‐item measure of identification on a seven‐point scale (see Table 2 for items and scale anchors). These six items covered various facets of social identification, including feelings of solidarity, cognitive centrality, and self‐stereotyping with the group (Ashmore *et al*., 2004; Leach *et al*., 2008), as well as Postmes, Haslam, and Jans's (2013) single‐item measure of identification. This scale showed good reliability (T0–T3: α = .85–.90).

**Table 2:** Social identification items

I feel loyal to this team
I often think about the fact that I am a member of this team
I have a lot in common with other team members
Being a member of this team is important to who I am
I feel committed to this team
I identify with this team

Note

All questions were rated on a 7‐point scale ranging from 0 to 6. Scale anchors were 0 = Strongly disagree, 3 = Neither agree nor disagree, 6 = Strongly agree.

Individuals’ perception of their own performance was measured using the following single item: ‘Irrespective of the result, how do you rate your *individual* performance’? Individual perceptions of team performance were recorded using the following single‐item question: ‘Irrespective of the result, how do you rate your *team* performance’? As a measure of actual performance, participants were asked to record the score of their last team match. Actual team performance was subsequently calculated as the score difference for each match (e.g., 3–1 loss would be recorded as −2). As these scores were identical for the whole team, actual performance was calculated only at the team level.^1^ These score differences were then standardized by creating *Z*‐scores for the score differences within each sport (Smith *et al*., 2001; Wolfe & Box, 1987).

Items were translated from English into Italian and then independently back‐translated by translators naïve to the aims of the study (Brislin, 1970). Original and back‐translated versions were compared, any discrepancies were discussed, and the translation was adjusted where necessary (Sireci, Yang, Harter, & Ehrlich, 2006).

## Results

Our analytic approach consisted of two phases. First, we sought to validate the construct of TLI by exploring intraclass correlations (ICC's), as well as trends in within‐team and between‐team variance in identification. Thus, we examined whether members of the same team become increasingly similar in their levels of identification over time (i.e., consensualization), as well as whether teams become increasingly different in their levels of identification (i.e., polarization). Our main analyses then investigated how ILI, TLI, and performance outcomes are related using multilevel cross‐lagged models. This allowed us to examine prospective, directional relationships between social identification and performance outcomes at both the individual and team level. We dealt with missing data by using full‐information maximum likelihood estimation (Allison, 2003) in Mplus 6.0 for all our analyses. Descriptive statistics are displayed in Table 3. Within‐team and between‐team zero‐order correlations for identification and performance outcomes are shown in Table 4.

**Table 3:** Means and standard deviations for team identification, perceived individual performance, perceived team performance, and actual team performance at each time point

	Time 0			Time 1			Time 2			Time 3		
	Mean	*SD*	ICC (%)	Mean	*SD*	ICC (%)	Mean	*SD*	ICC (%)	Mean	*SD*	ICC (%)
Team identification	4.36	0.90	16.0	4.30	0.96	26.2	4.25	0.99	33.6	4.28	1.06	38.9
Perceived individual performance	3.70	1.33	9.5	3.80	1.24	11.1	3.89	1.29	14.7	3.63	1.43	15.8
Perceived team performance	3.91	1.22	24.2	3.93	1.10	19.7	4.08	1.09	26.7	3.88	1.44	27.2
Actual team performance	−0.08	0.94		0.02	0.70		−0.01	1.06		−0.02	1.02	

Note

ICC's for identification and perceived performance outcomes are also shown.

**Table 4:** Within‐team (below diagonal) and between‐team (above diagonal) zero‐order correlations between team identification and performance outcomes at each time point

		1	2	3	4	5	6	7	8	9	10	11	12	13	14	15	16
1.	Team identification (T0)	–	.89	.82	.78	.25	.42	.74	.54	.31	.51	.80	.58	−.27	.17	.30	.28
2.	Team identification (T1)	.73	–	.94	.92	.31	.53	.71	.48	.30	.54	.83	.45	−.17	.24	.30	.22
3.	Team identification (T2)	.61	.72	–	.98	.21	.47	.74	.60	.28	.45	.82	.52	−.17	.14	.22	.38
4.	Team identification (T3)	.58	.67	.74	–	.20	.52	.73	.66	.30	.48	.81	.60	−.09	.21	.16	.34
5.	Perceived individual performance (T0)	.21	.19	.15	.27	–	.57	.41	−.00	.76	.40	.30	−.03	.33	.37	.32	.13
6.	Perceived individual performance (T1)	.16	.23	.18	.27	.43	–	.56	.51	.40	.75	.71	.43	.31	.56	−.02	.21
7.	Perceived individual performance (T2)	.02	.15	.17	.21	.18	.33	–	.67	.42	.36	.82	.58	−.37	.13	.01	.37
8.	Perceived individual performance (T3)	−.02	.04	.09	.18	.32	.37	.32	–	.09	.38	.60	.87	−.06	.38	−.11	.64
9.	Perceived team performance (T0)	.28	.23	.27	.30	.51	.31	.08	.15	–	.43	.28	.22	.44	.31	.18	.03
10.	Perceived team performance (T1)	.19	.29	.15	.27	.35	.58	.23	.16	.25	–	.65	.33	.39	.58	.28	.29
11.	Perceived team performance (T2)	.02	.18	.24	.26	.15	.28	.54	.19	.17	.28	–	.57	−.24	.24	.09	.27
12.	Perceived team performance (T3)	.05	.13	.18	.26	.13	.12	.20	.63	.16	.03	.21	–	−.02	.41	−.29	.33
13.	Actual team performance (T0)	–	–	–	–	–	–	–	–	–	–	–	–	–	.43	.04	.05
14.	Actual team performance (T1)	–	–	–	–	–	–	–	–	–	–	–	–	–	–	.15	.25
15.	Actual team performance (T2)	–	–	–	–	–	–	–	–	–	–	–	–	–	–	–	.17
16.	Actual team performance (T3)	–	–	–	–	–	–	–	–	–	–	–	–	–	–	–	–

### Validating TLI

#### Intraclass correlations

To explore the proportion of the variance in individuals’ responses to identification that can be attributed to the group or team level, ICC's of identification were examined. ICCs estimate the extent to which individuals within the same team are more similar in their levels of identification than are individuals in general (Hox, 2010). An ICC of 0 would show that individuals in the same team would be no more similar in their degree of identification than individuals in general, whereas an ICC of 1 would show that individuals in the same team are completely identical in their level of identification. Thus, the ICC represents the proportion of systematic team‐level variance in identification (i.e., the proportion of TLI to ILI). The ICC's for our data show that 16% of the total variation in identification can be attributed to the team at T0. Consistent with the idea of TLI as an emerging property of the team, the ICC appeared to increase over time to 26.2%, 33.6%, and 38.9% from T1 to T3, respectively (these ICC's are considered quite high for small groups, Hox, 2010). This apparent increase in ICC over time could be due to consensualization (members become increasingly similar in their levels of identification) and/or polarization (teams, as a whole, become increasingly different from one another in their levels of identification). To see which it was, we examined separately the two variance components used to calculate the ICC – within‐team variance (individual level) and between‐team variance (team level).

#### Within‐ and between‐team variances

A reduction over time in within‐team variance would indicate that the influence of the team on identification occurs as individuals in the same team become closer in their levels of identification over time (i.e., consensualization). On the other hand, the influence of the team on identification could also be the result of an increase in between‐team variance in the levels of identification over time (i.e., polarization).

**Figure 1:**
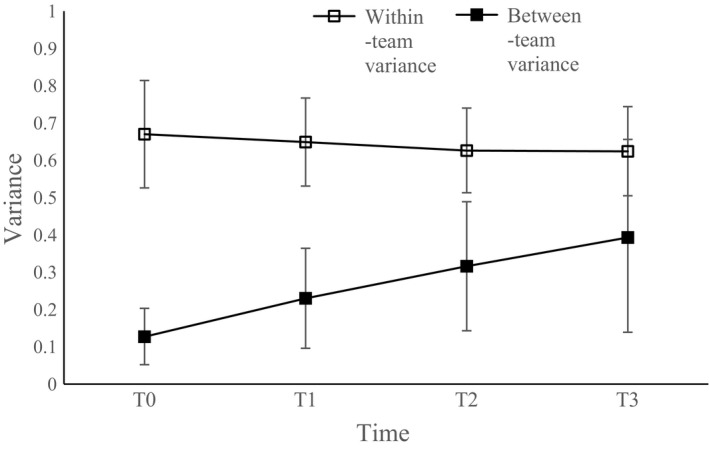
Within‐team and between‐team variance in social identification across four time points with 95% confidence intervals.

As shown in Figure 1, within‐team variance in identification appears to be stable over time, whereas between‐team variance appears to increase. To test this statistically, we first created a baseline model that allowed within‐team and between‐team variances in identification to be freely estimated. Next, we examined whether members of the same team became more similar in their levels of identification over time by constraining within‐team variance to be equal across all time points and comparing this to our freely estimated baseline model. Chi‐square difference testing, using the Satorra–Bentler scaled chi‐square (S‐B χ^2^: Bryant & Satorra, 2012), revealed that constraining within‐team variance to be equal across all time points did not significantly reduce model fit: Δ S‐B χ^2^ (3) = 0.673, *p *=* *.82. Further analyses showed that there were also no significant differences in the size of within‐team variance between adjacent time points (e.g., from T0 to T1 or from T2 to T3): all Δ S‐B χ^2^ (1) ≤ 0.447; all *p *≥* *.50. This suggests that the apparent increase in ICCs shown above is *not* the result of members of the same team becoming more similar in their levels of identification over time – that is, group consensualization.

Conversely, constraining the between‐team variance across all time points did significantly decrease model fit compared to our freely estimated baseline model: Δ S‐B χ^2^ (3) = 32.362, *p *<* *.001. Exploring this further, we tested the effects of constraining between‐team variance across pairs of adjacent time points. This showed a significant increase in between‐team variance from T0 to T1, Δ S‐B χ^2^ (1) = 49.696, *p *<* *.001, a marginal increase from T1 to T2, Δ S‐B χ^2^ (1) = 3.144, *p *=* *.076, and a significant increase from T2 to T3, Δ S‐B χ^2^ (1) = 8.00, *p *=* *.005.^2^ Thus, the influence of the team on identification (i.e., TLI) appears to be due to teams becoming increasingly different in their levels of identification over time – that is, group polarization.

### Predicting individual and team performance

**Figure 2:**
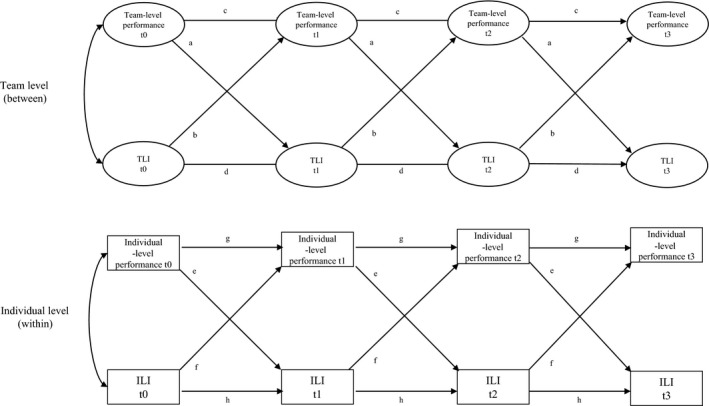
An example of a multilevel cross‐lag regression model for relations between social identification and performance at the team (between) and individual level (within) across four time points (t0–t3). For each level (team and individual), the relations between factors are specified as cross‐lag effects, which indicate the prospective effect of one variable on the other (e.g., the effect of TLI t0 on team‐level performance t1) after controlling for their stability across time (e.g., the autoregressive path of TLI t0 to TLI t1). The letters display constraints imposed on stability (autoregressive) and cross‐lagged coefficients across time. Residual covariances are included in the model, but are not shown in the figure to aid clarity.

Our main analyses examined prospective, directional relationships between social identification at each level (ILI and TLI) and performance (perceived individual, perceived team, and actual team performance), using multilevel cross‐lagged structural equation models (see Figure 2). We ran separate multilevel cross‐lagged models for perceived individual performance, perceived team performance, and actual team performance, controlling for country and sport type (i.e., ‘individual’ or team‐based).^3^ We accounted for variance due to specific measurement occasions by correlating residual variances within waves (e.g., the residual of TLI at Time 1 with the residual of team performance at Time 1). We also allowed identification and performance to covary freely at T0 at both levels of analysis. In order to gain statistical power and parsimony, the residual covariances and the effects of sport type and country were constrained to be equal at T1, T2, and T3. Similarly, the stability (autoregressive) and cross‐lagged coefficients were constrained to be equal across time (i.e., each T0 to T1 path was constrained to be equal to the corresponding T1 to T2 path and the corresponding T2 to T3 path). This gave us one parameter, instead of three, to test each of the predicted effects.

This analytic approach allows us to compare individual‐ and team‐level effects of social identity on performance, as well as vice versa. However, it should be noted that, since there are fewer teams (*N *=* *45) than individuals (*N *=* *369), a larger effect size is required for team‐level parameters to achieve statistical significance. Fit was assessed by the comparative fit index (CFI, good fit >.95, acceptable fit >.90), root mean square error of approximation (RMSEA, good fit <.06, acceptable fit <.08), and standardized root mean square residual (SRMR, good fit <.08, acceptable fit <.10, see Hu & Bentler, 1999; Kline, 2005).

As shown in Table 5, fit indices for all three models were acceptable. Table 5 also reports the estimates for the autoregressive and cross‐lagged coefficients.^4^ Supporting our hypotheses, team‐level variation in perceived team performance (H1) and actual team performance (H2) were both prospectively predicted by TLI, and team‐level variation in perceived individual performance was marginally predicted by TLI (H3). Notably, all of these effects were unidirectional, with team identification predicting subsequent performance but performance not predicting subsequent team identification.^5^

**Table 5:** Cross‐lagged and autoregressive effects of performance and social identification

Model	Level	Cross‐lagged effects								Autoregressive effects								Fit indices		
		SI **→** P				P **→** SI				SI **→** SI				P **→** P				CFI	RMSEA	SRMR
		*B*	*SE*	*p*	β	*B*	*SE*	*p*	β	*B*	*SE*	*p*	β	*B*	*SE*	*p*	β			
Perceived individual performance	WT	0.09	.05	.087	.06	0.02	.02	.341	.03	0.71	.03	<.001	.72	0.33	.06	<.001	.35	.913	.064	.085
	BT	0.46	.27	.088	.51	0.01	.11	.951	.01	1.05	.10	<.001	.86	0.49	.26	.063	.64			.096
Perceived team performance	WT	0.13	.05	.016	.10	0.01	.02	.594	.02	0.72	.03	<.001	.72	0.23	.06	<.001	.25	.950	.047	.067
	BT	0.92	.31	.003	.73	−0.03	.06	.593	−.05	1.08	.10	<.001	.89	0.14	.19	.473	.18			.075
Actual team performance	WT	–	–	–	–	–	–	–	–	0.72	.03	<.001	.72	–	–	–	–	.959	.046	.082
	BT	0.82	.25	.001	.42	0.00	.03	.967	.00	1.04	.08	<.001	.86	0.18	.12	.121	.23			.060

Notes

Unstandardized paths (*B*) were constrained to be time‐invariant (i.e., T0→T1 = T1→T2 = T2→T3). Standardized paths (β) are reported here for T0→T1 paths.

BT = between‐team; P = Performance; SI = Social Identification; WT = within‐team.

As shown in Table 5, ILI significantly predicted individuals’ perceptions of team performance and (marginally) individual performance. However, these effects were considerably smaller than the corresponding effects at the team level, consistent with our prediction that the effects of TLI on team performance would not be reducible to aggregated effects of ILI on individual performance (H4). The estimated slope of team performance on identification at the team level (0.92) was more than seven times that of the corresponding slope at the individual level (0.13), and the 95% confidence intervals for the two slopes did not overlap (individual level: 0.02, 0.23; team level: 0.32, 1.53). We tested whether model fit decreased once the paths from ILI and TLI to perceived team performance were constrained to be equal, which would be the case if effects of TLI were reducible to aggregated effects of ILI. Chi‐square difference testing using the Satorra–Bentler scaled chi‐square (Bryant & Satorra, 2012), indicated a non‐significant loss of fit, Δ S‐B χ^2^ (1) = 2.54, *p *=* *.11; however, the constrained model showed an unacceptable value of SRMR_between_ (.148), indicating that it poorly represented team‐level relationships in the data. Thus, the adequately fitting model with separate effects of TLI and ILI should be preferred, supporting H4.

For perceived individual performance, the estimated slope for TLI (.46) was over five times the estimated slope for ILI (.09); however, since both effects were marginal their 95% confidence intervals overlapped (individual level: −0.01, 0.18; team level: −0.07, 0.98). We tested whether model fit decreased once the paths from ILI and TLI to perceived team performance were constrained to be equal. Chi‐square difference testing again indicated a non‐significant loss of fit, Δ S‐B χ^2^ (1) = 0.74, *p *=* *.39; however, the constrained model again showed an unacceptable value of SRMR_between_ (.155). Thus, the adequately fitting model with separate effects of TLI and ILI was preferred – providing qualified support for H4, since effects of both TLI and ILI on individual performance reached only marginal significance.

## Discussion

Supporting our main hypotheses, TLI predicted perceived (H1) and actual team performance (H2). We also found that TLI marginally predicted systematic team‐level variance in individual performance ratings (H3). Thus, our findings show a consistent picture: TLI was empirically separable from ILI and prospectively predicted perceived and actual team performance. Equally, ILI only marginally predicted perceived individual performance, and performance does not predict ILI or TLI. Our results also validate the construct of TLI by showing that the influence of the team on identification became stronger over time. We further show that this effect was due to group polarization, rather than group consensualization, indicating that teams became increasingly different in their levels of identification over time. Taken together, these findings support calls for a multilevel interpretation of social identification and highlight the significant influence of shared social identification on group‐level processes and outcomes, over and above its individual‐level effects.

### Theoretical implications

By treating social identity as a multilevel construct, we have demonstrated the considerable differences in how TLI and ILI can influence performance. Previous research, that has tended to ignore TLI, may have drawn misleading conclusions regarding the effect of ILI on performance (Riketta & Dick, 2005). Although these findings diverge from the current ILI performance landscape, they are nevertheless in accordance with social identity predictions (Ellemers, De Gilder, & Haslam, 2004). As argued by Ellemers *et al*., high levels of shared identification will cause team members to strive to achieve team‐rather than individually orientated performance goals. It follows that, as long as performance is a goal, high TLI will cause high levels of team performance (Dietz *et al*., 2015; Haslam, 2004; van Knippenberg, 2000). Equally, a team with a strong TLI may benefit from improved team environments that facilitate training, engagement, and ultimately performance. Seen in this way, the influence of TLI on team performance outcomes appears to be due to team‐level processes. Exploring exactly how TLI influences team‐level performance outcomes may be a fruitful avenue for future research.

Team influence on identification can either be the result of a process of ‘consensualization’ in which group members come to agree on the characteristics of the team and in their experiences of team membership, or it can be the result of a process of ‘polarization’ in which teams become increasingly different from each other in characteristics and experiences. Our results suggest teams do indeed become increasingly different in their levels of identification (i.e., group polarization), indicating that some teams are able to build and foster a stronger sense of identification than others. Over time, these teams appear to be able to develop a shared narrative and knowledge of what it means to be part of that team (Kerr *et al*., 2000; Postmes *et al*., 2005). The reasons why some teams are able to foster a strong sense of identity, while others are not, could be due to a number of factors that occur across the whole team, such as team climate or effects of leadership (Fransen *et al*., 2015; Kerr & Hertel, 2011; van Dick *et al*., 2006). Investigating these effects on the development of TLI will be an important avenue for future research.

Overall, our findings represent a growing movement towards a more complete interpretation of social identity processes (Jans *et al*., 2015; Ozeki, 2015). Lack of attention to group‐level effects is likely to lead to an overestimation of individual‐level effects (Hoffman, 2015; Hox, 2010). This may inadvertently reinforce the view that group identification is solely an individual difference variable. Equally, focusing only on measurement at the group level ignores the potentially important variance among individuals within each group that may explain their behaviour. Thus, whenever psychological processes operate at multiple levels of analysis, it is important to examine these phenomena with a multilevel approach. While it falls beyond the scope of this paper to speculate on how a multilevel approach to social identity may alter understandings of other behavioural outcomes, we urge future researchers to consider carefully the multilevel facets of social identity processes.

### Practical implications

Given that TLI seems to impact performance, and social identification is considered highly malleable (Onorato & Turner, 2004), targeting TLI could be an important strategy for leaders, coaches, and team building facilitators. One possible approach would be to harness social identity motives, such as a sense of collective continuity, as they have been shown to predict social identification (Thomas *et al*., 2017). Facilitated team‐level discussions could then be used to target and attempt to increase satisfaction of those motives that are poorly satisfied. For instance, if a team‐level evaluation illustrated that a team has a poor sense of continuity, the team should focus on discussions and strategies for increasing continuity for the whole team. According to this proposition, increasing satisfaction of social identity motive(s) across the whole team will lead to an increase in TLI, which will in turn lead to an increase in team performance. Since team‐orientated performance outcomes are often more important than individual ones (Salas, Cooke, & Rosen, 2008), such interventions may be particularly important in organizational, as well as sporting, settings.

### Research strengths and limitations

The current research has several notable strengths. Our multilevel and longitudinal design has enabled us to draw conclusions regarding the influence of individual‐ and team‐level effects. Notwithstanding the reduction in power at the team level (i.e., smaller number of teams than individuals, *N *=* *369 individuals, *N *=* *45 teams), the influence of TLI is particularly notable and demonstrates the potential for team‐level effects to influence behaviour. This methodology appears crucial to the study of teams and groups in general, and we strongly encourage those conducting future research in this area to take a similar approach.

One possible limitation is that not all of the sports in our sample were ‘team sports’ (e.g., tennis, badminton, and swimming). However, even in these ‘individual’ sports, the athletes still behaved as teams. They went on social activities together, trained together, attended matches together, and, most crucially, won and lost *as a team,* rather than individuals. Thus, given that TLI is likely to influence effects such as effort and attendance to training and matches, it is also likely to affect performance – whether or not performance is directly attributable to the team. Note also that many of these ‘individual’ sports typically also included team‐based performance tasks (e.g., doubles teams in tennis and relays in swimming).

Another possible limitation with our sample is that our findings may not be applicable to teams in different environments. For example, Jans *et al*. (2015) found that group identity in online groups was based mainly on individual representations of the group (i.e., ILI). Equally, the development of identification within large social categories is distinctly different from identity processes that occur in team situations. Identification in social categories typically emerge based on cognitively shared abstractions about group norms and values, while team identification has mostly been shown to emerge from face‐to‐face interactions and common goals (Deaux & Martin, 2003; Easterbrook & Vignoles, 2012; Serpe & Stryker, 2011; van Veelen, Eisenbeiss, & Otten, 2016; Wheelan, 2005). Thus, our findings may not be transferable to teams that do not interact on a regular basis (e.g., virtual teams, see Gibson & Cohen, 2003) or larger social categories. Nevertheless, our sampling of sports teams did span 14 different sports across two countries and therefore should have some generality to other small group environments where team members interact on a regular basis.

### Concluding remarks

Our main finding that TLI predicts perceived and actual team performance – and that it does so over and above possible aggregated effects of ILI – embodies a much needed movement towards more team‐level (or, more generally, group‐level) research within the social identity literature (Jans *et al*., 2015; Ozeki, 2015). This research also speaks to the original group‐level spirit of the social identity approach and serves as an important reminder that humans operate as part of a complex social organization with higher order frames of reference. Our hope is that future research further establishes TLI as a construct and that this leads to teams fostering TLI and improving team performance. As teams and groups form the foundations of our society, taking this small step could have positive impacts on an array of sporting, organizational, and other collective ventures.

## Footnotes

1 There were a few discrepancies in actual performance scores that participants recorded, whereby a team member recorded a different score from the rest of the team. In these situations, the majority score (i.e., mode) was used.

2 The weaker increase from T1 to T2 coincided with the Christmas holidays.

3 Fencing, trampolining, badminton, tennis, swimming, and cycling were coded as ‘individual’ sports. Hockey, football, volleyball, ultimate frisbee, basketball, netball, water polo, and synchronized swimming were coded as ‘team’ sports. We controlled for type of sport and country by including these two binary variables as predictors of between‐level variance in identification and performance at T0–T4.

4 Although the coefficients were constrained to be equal across time intervals, the constraints were imposed on unstandardized coefficients (Kenny, 2005), and so there are slight variations over time in the resulting standardized coefficients.

5 Because social identification was a scale and performance ratings were single items, one could argue that this effect is driven because the scale creates a more stable construct over time (as displayed by the higher regression coefficients for the autoregressive paths). This leaves less variance to be explained by the lagged relationship from performance to identity than there is to be explained by the lagged relationship from identity to performance. To rule out this alternative interpretation, we used Postmes *et al*. (2013) single‐item measure of social identification and reran the analyses. The patterns of significant findings involving perceived and actual team performance remained consistent, giving us confidence in our main findings. In contrast, marginal effects involving perceived individual performance were reduced to non‐significance in these alternative analyses.
